# Bibliographical

**Published:** 1868

**Authors:** 


### BIBLIOGRAPHICAL.

A PRACTICAL TREATISE ON THE DISEASES OF WOMEN. By T. GAILLARD THOMAS, M. D., *Professor of Obstetrics and the Diseaees of Women and Children in the College of Physicians and Surgeons, New York; Physician to Bellevue Hospital; Consulting Physician to the State Women's Hospital; Late President of the New York Obstetrical Society; Member of the New York Academy of Medicine; of the County Medical Society, &c., &c.* With Two Hundred and Nineteen Illustrations. Philadelphia: Henry C. Lea. 1868.

We have only had time to give this new volume of 625 pages, upon a very important class of diseases, a hasty examination, from which we are led to believe that it is the best work that has yet appeared on the diseases of women. There is about it a precision and accuracy, a fullness and completeness, a clearness and simplicity, to be found in no other book on the same subject within our knowledge. It steers clear of the hobbies of Bennett, Simpson, Tyler Smith, Tilt, and others; its views on uterine pathology are, to our mind, more consonant with reason and common sense than any other we have seen. Enjoying fine opportunities in an extensive field of observation, Dr. Thomas, as an author, has fully sustained his high reputation as a teacher.

We may refer to this work again after a more thorough examination of its contents, and in the meantime, have no hesitation in strongly recommending it to the profession as the best exposition yet published of the subjects of which it treats.

